# Ultra-low-loss and large-effective-area fiber for 100 Gbit/s and beyond 100 Gbit/s coherent long-haul terrestrial transmission systems

**DOI:** 10.1038/s41598-019-53381-1

**Published:** 2019-11-20

**Authors:** Dong Wang, Yunbo Li, Dechao Zhang, Hongyan Zhou, Yang Zhao, Lei Wang, Rui Tang, Xin Zhao, Lei Zhang, Jun Wu, Ruichun Wang, Jie Luo, Wenyu Zhao, Han Li

**Affiliations:** 1 Department of Network and IT Technology, Research Institute of China Mobile, Beijing, 100053 China; 2 State Key Laboratory of Optical Fiber and Cable Manufacture Technology, Yangtze Optical Fiber and Cable Joint Stock Limited Company, Wuhan, 430073 China; 3 China Academy of Information and Communication Technology, Beijing, 100191 China

**Keywords:** Fibre optics and optical communications, Nonlinear optics

## Abstract

Ultra-low-loss and large-effective-area fiber has been successfully applied in transoceanic transmission, which is considered as a promising candidate for 100 Gbit/s and beyond 100 Gbit/s coherent long-haul terrestrial optical networks. Several theoretical and experimental investigations have been reported, including provincial terrestrial field trial. To support long-haul terrestrial application, it is urgent to prove that the ultra-low-loss and large-effective-area fiber after terrestrial deployment can significantly enhance the performance of long-haul transmission over 1000 km compared with the conventional single mode fiber. In this paper, we extended our previous work and summarized design methods for complex terrestrial environment. To verify the fiber characteristics in long-haul terrestrial transmission, we installed the longest terrestrial ultra-low-loss and large-effective-area fiber link in the world with a total length of 1539.6 km. The results show that the transmission performances of wavelength-division-multiplexed signals with per-channel data rates of 100 Gbit/s, 200 Gbit/s, and 400 Gbit/s over the ultra-low-loss and large-effective-area fiber are all obviously improved, demonstrating that this fiber is more suitable for ultrahigh-speed long-haul terrestrial transmission.

## Introduction

With the rapid development of advanced services, such as mobile internet, high-definition video, cloud computing, virtual reality, etc., the demand for the capacity of existing 100 Gbit/s coherent optical transport network exhibits a significant increasing trend. Single-carrier 200 Gbit/s and 400 Gbit/s coherent technologies are introduced to further increase the transmission capacity^1–4^. However, coherent transmission systems with per-channel data rates beyond 100 Gbit/s are facing enormous challenges on improving unrepeatered transmission distance. Several schemes have been proposed, such as using probability constellation shaping^5^, Raman amplification^6^, new-type optical fiber^7^, etc. For coherent optical transmission system, the transmission performance is mainly limited by the attenuation and nonlinear effects in fiber as the dispersion can be sufficiently compensated by digital coherent detection^8^.

With the development of optical fiber technology, novel optical fibers have been achieved, such as ultra-low-loss fiber^9,10^, large-effective-area fiber^11,12^, etc. The optical signal-to-noise ratio (OSNR) after transmission over the same fiber link could be improved by reducing the link loss. Additionally, the optimum optical power before launching into the fiber link can be increased as the effective area of fiber is enlarged, which is also benefit for improving the received OSNR. Therefore, the optical fiber with a combination of low attenuation coefficient and large effective area may be a promising candidate to optimize the transmission performance for next-generation ultrahigh-speed long-haul optical network, which have been performed and applied for transoceanic submarine cable system^13,14^, and is specified by Recommendation ITU-T G.654. However, the characteristics of the installed optical fiber for terrestrial transmission are quite different from that of submarine cable, including macro-bending loss, operating temperature, etc. ITU-T introduced a new sub-category of the ultra-low-loss and large-effective-area fiber subsequently to support 100 Gbit/s and beyond 100 Gbit/s coherent terrestrial transmission, which is named as G.654.E fiber. Several design methods and lab tests of the G.654.E fiber are presented^15–19^. Recently, the results of a field trial with an installed 430-km G.654.E fiber link are reported^20^. Considering that the planning and construction of a long-haul optical fiber link usually last 2–3 years, it is urgent to decide which type of optical fiber is more suitable for future 100 Gbit/s and beyond long-haul transmission. However, the performance of the G.654.E fiber has not been verified in long-haul terrestrial transmission beyond 1000 km.

In this study, we introduced and summarized the design and characteristics of the ultra-low-loss and large-effective-area fiber. The macro-bending loss and operating temperature tests are carried out, demonstrating that the requirements of the complex terrestrial environment are fully satisfied. To evaluate the long-haul transmission performance of the fiber, a 22-span 1539.6-km terrestrial transmission system is deployed, which is the world’s longest terrestrial optical link based on the ultra-low-loss and large-effective-area fiber. Through the field trial, the advantages of this novel fiber are entirely exhibited, such as low attenuation coefficient, more robust to nonlinear effects, etc. Compared with the standard single mode fiber link, the OSNR margin of 100 Gbit/s PM-QPSK, 200 Gbit/s PM-QPSK, 200 Gbit/s PM-16QAM, and 400 Gbit/s PM-16QAM signals after transmission over the ultra-low-loss and large-effective-area fiber link with the same length can be improved by 2.39 dB, 2.76 dB, 1.82 dB, and 1.87 dB, respectively. With 1-dB OSNR penalty, the launch power of these signals are all improved about 1 dB. This is the first demonstration of the deployed ultra-low-loss and large-effective-area fiber for long-haul terrestrial transmission beyond 1000 km which provides convincing proof to the whole industry chain that the ultra-low-loss and large-effective-area fiber is the most promising medium for 100 Gbit/s and beyond 100 Gbit/s coherent long-haul terrestrial transmission systems.

## Results

**Figure 1:**
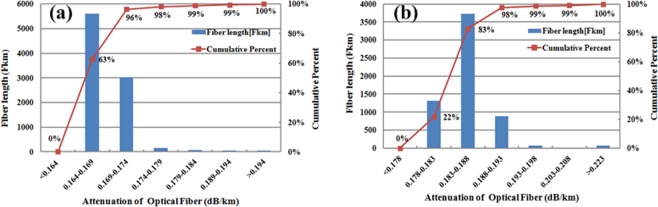
Attenuation distribution. The attenuation distribution of (**a**) the deployed G.654.E fiber and (**b**) the deployed G. 652.D fiber, respectively.

**Figure 2:**
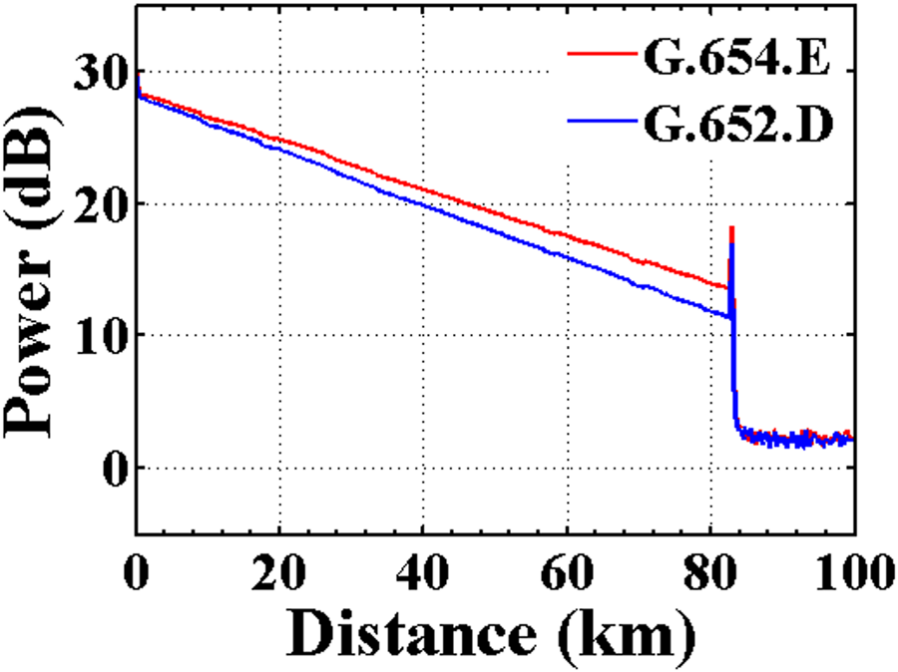
OTDR trace. The typical OTDR traces of the G.654.E fiber and the G.652.D fiber installed in the same cable.

The fiber characteristics of the installed 22-span 1539.6-km terrestrial transmission system are first evaluated. The ultra-low-loss and large-effective-area fiber, and the standard single mode fiber are installed in the same cable, which are briefly presented as the G.654.E fiber and the G.652.D fiber, respectively. The attenuation of 9011-Fkm G.654.E fiber link and 6093-Fkm G.652.D fiber link in this field trial are bi-directional measured by an optical time domain reflectometer (OTDR). Figure 1(a,b) show the frequency distribution of the fiber loss for the deployed G.654.E fiber link and G.652.D fiber link, respectively. The average attenuation coefficients of the optical fibers are 0.169 dB/km and 0.186 dB/km, respectively, which are highly consistent with the requirements of the corresponding specification. The typical measured OTDR traces of the G.654.E fiber and the G.652.D fiber installed in the same 82.96-km cable are shown in Fig. 2. We can see that the attenuation of the backscattered power is much lower along with the G.654.E fiber than that with the G.652.D fiber. Compared with the G.652.D fiber, the measured attenuation coefficient of the G.654.E fiber is reduced by about 0.017 dB/km, demonstrating the ultra-low-loss property of the deployed G.654.E fiber.

**Figure 3:**
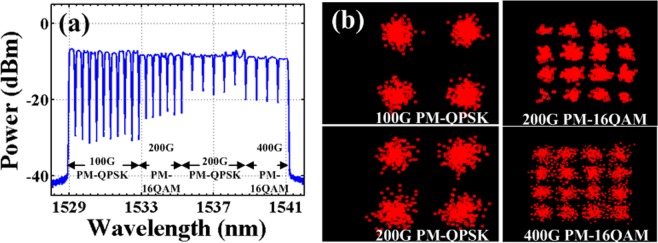
The optical spectrum and the constellation diagrams. (**a**) The optical spectrum after wavelength division multiplexing at the transmitter. (**b**) The constellation diagrams of the transmitted signals based on 100 Gbit/s PM-QPSK, 200 Gbit/s PM-16QAM, 200 Gbit/s PM-QPSK, and 400 Gbit/s PM-16QAM modulation, respectively.

The transmission performances of 100 Gbit/s and beyond 100 Gbit/s coherent optical systems over the G.652.D fiber and the G.654.E fiber are subsequently verified. Figure 3(a) shows the optical spectrum at the transmitter. Here, 100 Gbit/s PM-QPSK signals, 200 Gbit/s PM-16QAM signals, 200 Gbit/s PM-QPSK signals, and 400 Gbit/s PM-16QAM signals are multiplexed by wavelength selective switch (WSS). It is noted that these date rates are briefly stated and the actual date rates with FEC are 136 Gbit/s, 272 Gbit/s, 272 Gbit/s, and 544 Gbit/s, respectively. The channel spacing and channel number are consistent with that shown in Table 1. The corresponding constellation diagrams of the transmitted signals with different modulation formats are shown in Fig. 3(b), respectively. The multiplexed signals are then amplified by an Erbium doped fiber amplifier (EDFA) and launched into the optical fiber.

**Table 1:** The characteristics of 100 Gbit/s and beyond 100 Gbit/s optical signals.

	100 Gbit/s	200 Gbit/s		400 Gbit/s
Modulation Format	DP-QPSK	DP-16QAM	DP-QPSK	DP-16QAM
Channel Spacing	50 GHz	50 GHz	75 GHz	75 GHz
Channel Number	10	6	6	4

**Figure 4:**
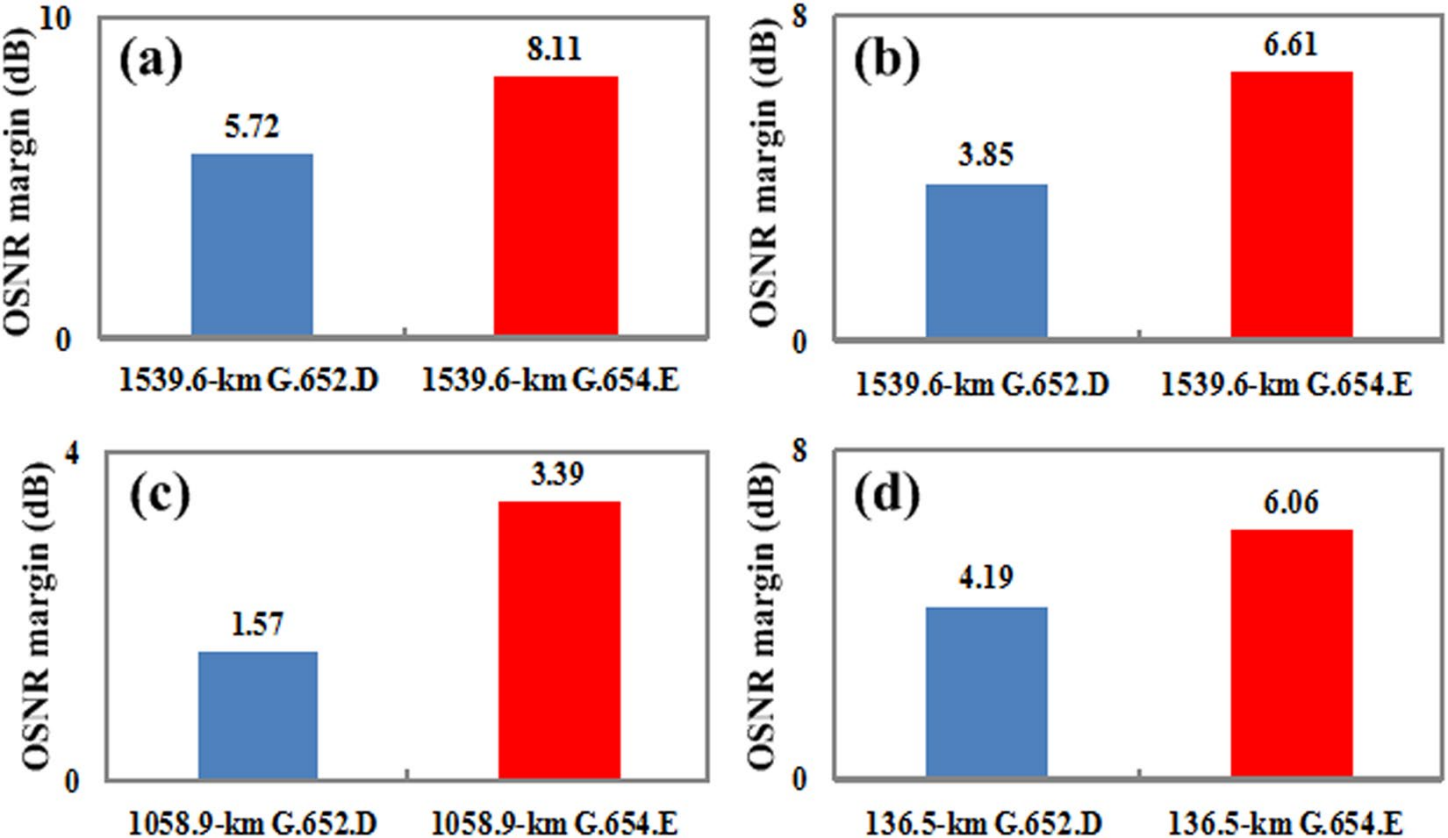
The OSNR margin at the receiver. The OSNR margin of (**a**) 100 Gbit/s PM-QPSK signal, (**b**) 200 Gbit/s PM-QPSK signal, (**c**) 200 Gbit/s PM-16QAM signal, and (**d**) 400 Gbit/s PM-16QAM signal after transmission over the G.652.D fiber and the G.654.E fiber, respectively.

The OSNR margin of the received signals with different modulation formats are shown in Fig. 4. As shown in Fig. 4(a), the OSNR margin of 100 Gbit/s PM-QPSK signal after transmission over a 22-span 1539.6-km G.654.E fiber can be improved by 2.39 dB than that over G.652.D fiber with the same length. Compared with G.652.D fiber link, the OSNR margin of 200 Gbit/s PM-QPSK signal and 200 Gbit/s PM-16QAM signal based on G.654.E fiber can be increased by 2.76 dB and 1.82 dB, which are shown in Fig. 4(b,c), respectively. Figure 4(d) shows that the OSNR margin of 400 Gbit/s PM-16QAM signal is increased from 4.19 dB to 6.06 dB as the G.654.E fiber is used instead of the G.652.D fiber. We can see that the transmission performances of 100 Gbit/s and beyond 100 Gbit/s signals are all improved significantly by using the G.654.E fiber.

**Figure 5:**
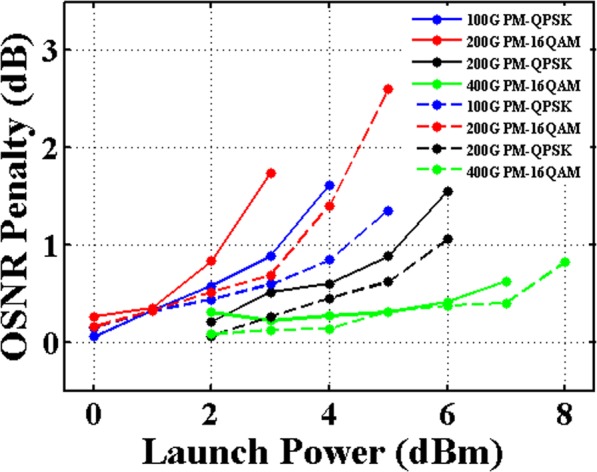
OSNR Penalty versus launch power. Solid lines represent the OSNR penalty after transmission over the G.652.D fiber link. Dash lines represent the OSNR penalty after transmission over the G.654.E fiber link.

The robustness in regarding to the nonlinear effects in the fibers is further investigated. The OSNR can be significantly improved by increasing the launch power of optical signals. However, when the input optical power beyond a certain value, the signal quality is degraded due to the enhanced nonlinear effects. Figure 5 shows the OSNR penalty of different modulation formats after transmission as a function of the launch power. Here, Post-FEC BER with 1.0E-12 is used for assessing the OSNR penalty. With 1-dB OSNR penalty, the optical power of 100 Gbit/s PM-QPSK signals, 200 Gbit/s PM-16QAM signals, 200 Gbit/s PM-QPSK signals, and 400 Gbit/s PM-16QAM signals before launching into the fiber are all improved about 1 dB by using the G.654.E fiber instead of the G.652.D fiber. The results indicate that the G.654.E fiber is more robust to the optical nonlinearity.

**Figure 6:**
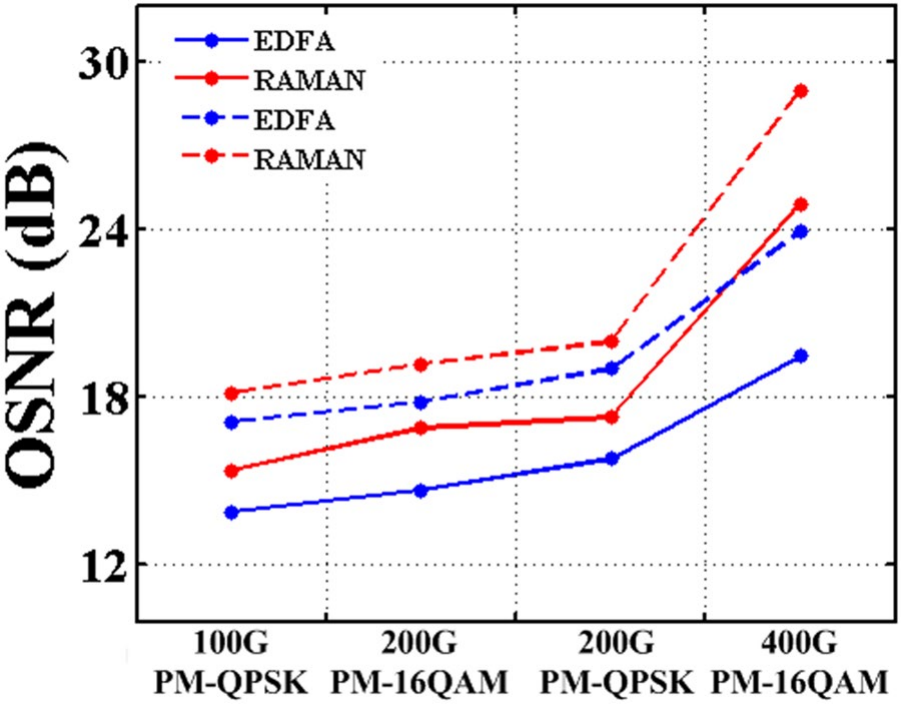
The OSNR after transmission by EDFA or Raman amplifier. Solid lines represent transmission over the G.652.D fiber link. Dash lines represent transmission over the G.654.E fiber link.

The influence of the effective area of the optical fibers on Raman amplification is also studied. Figure 6 shows the OSNR of different modulation formats after transmission over the G.654.E fiber and the G.652.D fiber with EDFA or Raman amplifier. Here, the two spans of the optical link between the node A and the node B with a total length of 136.5 km are replaced by a single span with the same length. A Raman amplifier or an EDFA is placed at the receiving side of this 136.5-km span. The rest of the 1539.6-km optical link remains unchanged by using EDFAs. As shown in Fig. 6, by using a Raman amplifier instead of the EDFA in the first span, the OSNR of the 100 Gbit/s PM-QPSK signals, 200 Gbit/s PM-16QAM signals, 200 Gbit/s PM-QPSK signals, and 400 Gbit/s PM-16QAM signals after transmission over the G.652.D fiber are improved about 1.5 dB, 2.2 dB, 1.5 dB, and 5.4 dB, respectively. For the G.654.E fiber link, an improvement of 1 dB, 1.4 dB, 1 dB, and 5 dB for the OSNR of the received signals with different modulation formats also can be observed, respectively. The difference of the improved OSNR by Raman amplification between the G.654.E fiber and the G.652.D fiber is from 0.4 dB to 0.8 dB. It is noted that Raman amplification is weakened as the effective area of the optical fiber is increased. The results show that Raman amplification is only slightly affected by using this G.654.E fiber instead of the G.652.D fiber.

## Conclusions

In this work, the world’s longest ultra-low-loss and large-effective-area fiber link for long-haul terrestrial transmission systems is performed. Many impressive properties of the ultra-low-loss and large-effective-area fiber are demonstrated through this field trial, such as low attenuation coefficient, more robust to nonlinear effects, etc. The results show that the transmission performances of 100 Gbit/s and beyond 100 Gbit/s coherent optical systems can be significantly improved by using this ultra-low-loss and large-effective-area fiber instead of the standard single mode fiber.

## Methods

### Design and manufacture of the ultra-low-loss and large-effective-area fiber

The fiber loss is composed of Rayleigh scattering loss, material absorption, macro-bending loss, etc. Here, Rayleigh scattering contributes to fiber loss dominantly. Thus, the fiber loss could be obviously decreased by using pure Si in fiber core. Moreover, the effective area can be further enlarged with the increase of the diameter of the fiber core. However, the increased effective area should result in the deterioration of the macro-bending loss. Compared with the submarine application, terrestrial transmission is more sensitive to the macro-bending loss. The trench assisted structure could be used to obtain the bending resistance performance. In this way, an ultra-low-loss and large-effective-area fiber with an attenuation coefficient of 0.17 dB/km, an effective area of 110 μm², and a macro-bending loss of 0.05 dB is achieved. Furthermore, the operating temperature range of the fiber also should be considered because the temperature on land varies greatly. In temperature cycling test, the fluctuation of the fiber attenuation is less than 0.01 dB/km as the temperature is changed from −40 °C to +70 °C, indicating that the ultra-low-loss and large-effective-area fiber can meet the requirements of the complex terrestrial environment.

### Configuration of the field trial

**Figure 7:**
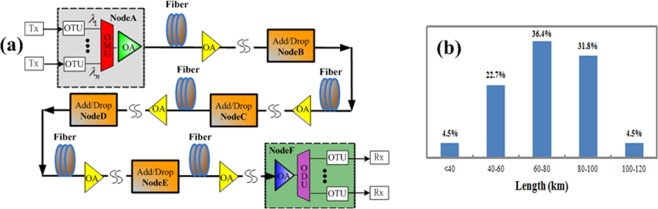
Configuration of the field trial. (**a**) The network topology of the 1539.6-km long-haul terrestrial transmission system. (**b**) The frequency distribution of span length for the 22-span fiber link.

A 22-span 1539.6-km G.654.E fiber is deployed in field trial to evaluate its performance for 100 Gbit/s and beyond 100 Gbit/s coherent long-haul terrestrial transmission. To exhibit the advantages of the G.654.E fiber, a G.652.D fiber is also installed in the same cable. The characteristics of the fibers are shown in Table 2. Figure 7(a) shows the network topology of the 1539.6-km long-haul terrestrial transmission system. Single-carrier 100 Gbit/s, 200 Gbit/s, and 400 Gbit/s optical signals centered at different wavelengths are combined and transmitted at node A. The other nodes are used to support wavelength add/drop or pass-through. The modulation format, channel spacing, and channel number of 100 Gbit/s and beyond 100 Gbit/s optical signals are shown in Table 1, respectively. Figure 7(b) shows the frequency distribution of span length for the 22-span fiber link. The longest and the shortest length of the fiber span are 102 km and 30 km, respectively. It is noted that the signal quality could be significantly degraded after transmission over a long-span fiber link.

**Table 2:** The characteristics of the G.654.E fiber and the G.652.D fiber.

	G.654.E	G.652.D
Effective Area @1550 nm	110 μm²	80 μm²
Attenuation @1550 nm	≤0.17 dB/km	≤0.19 dB/km
Macro-bending Loss (R30 mm × 100 turns) @1625 nm	≤0.05 dB	≤0.05 dB

### Performance simulation

Corresponding theoretical analysis and numerical calculation based on the configuration shown in Fig. 7 are subsequently carried out to verify the transmission performance by using different fibers. When optical signals propagate through a fiber, the process can be described by the nonlinear Schrödinger equation^21^

1 $$\frac{\partial U}{\partial z}+\frac{\alpha }{2}U+\frac{i{\beta }_{2}}{2}\frac{{\partial }^{2}U}{\partial {t}^{2}}-\frac{{\beta }_{3}}{6}\frac{{\partial }^{3}U}{\partial {t}^{3}}=i\gamma [{|U|}^{2}U+\frac{i}{{\omega }_{0}}\frac{\partial }{\partial t}({|U|}^{2}U-{t}_{R}U\frac{\partial {|U|}^{2}}{\partial t})]$$

where *U* and *ω*_0_ are the complex envelope and central angular frequency of the optical signal, respectively; *α* is the fiber loss; *β*_2_ and *β*_3_ are the dispersion parameter; *t*_*R*_ is related to stimulated Raman scattering; *γ* is the nonlinear coefficient, which can be expressed as^21^

2 $$\gamma =\frac{{n}_{2}{\omega }_{0}}{c{A}_{eff}}$$

where n_2_ is the nonlinear-index coefficient; *c* is the transmission speed of light in vacuum; *A*_*eff*_ is the effective area of fiber. By using coherent detection, dispersion compensation can be effectively achieved at the receiver. Therefore, the fiber loss and the nonlinear coefficient are the most crucial parameters for coherent optical transmission. As shown in Eq. 2, the nonlinear coefficient *γ* is inversely proportional to *A*_*eff*_. It means that the nonlinear transmission impairments can be suppressed by enlarging the effective area of fiber. However, there is a compromise between the effective area and the macrobending loss for optical fiber design as the macrobending loss can be enhanced with the increase of the effective area of fiber. According to the above analysis, the ultra-low-loss and large-effective-area fiber is more suitable for coherent long-haul transmission.

**Figure 8:**
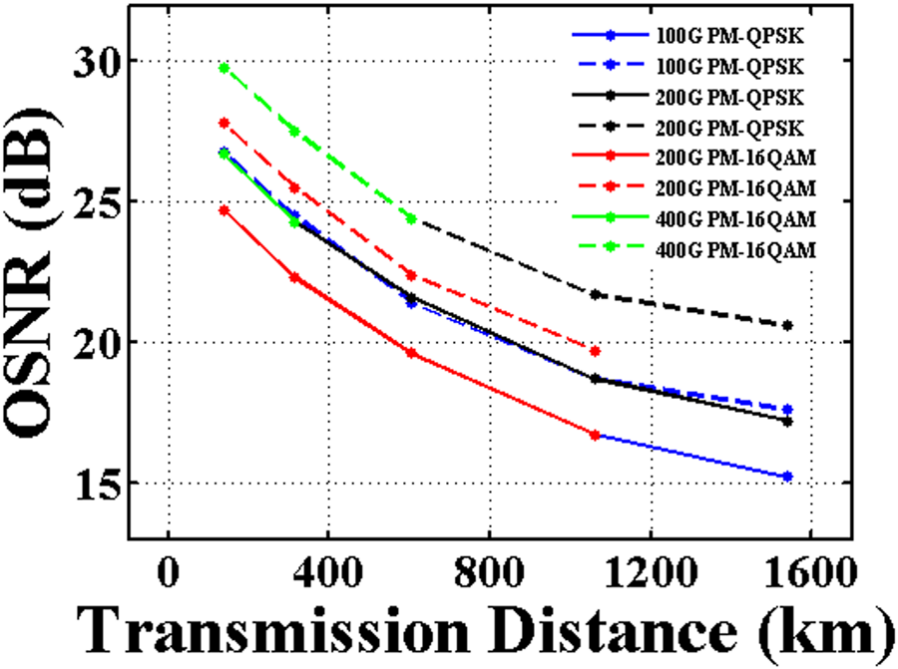
OSNR versus transmission distance. Solid lines represent the OSNR after transmission over the G.652.D fiber link. Dash lines represent the OSNR after transmission over the G.654.E fiber link.

In the following simulation, the optical parameters of optical transmitter, optical amplifier, and optical fiber are highly consistent with that used in the field trial, which can be practically achieved. Figure 8 shows the OSNR of 100 Gbit/s and beyond 100 Gbit/s optical signals as a function of the transmission distance. Due to the accumulated amplified spontaneous emission (ASE) noise, the OSNR of optical signals with different modulation formats after transmission are all significantly degraded. It also can be seen that the OSNR of the optical signals transmitted over G.654.E fiber is obviously improved compared with transmission over G.652.D fiber, demonstrating that the G.654.E fiber is benefit for coherent long-haul transmission. We attributed the improvement to the enhanced optical launch power as the G.654.E fiber is more robust to fiber nonlinearity.

## Data Availability

The data used to support the findings of this study are available from the corresponding author upon request.
